# Assessment of hospitalizations in schizophrenia patients treated with paliperidone 1-monthly (PP1M), paliperidone 3-monthly (PP3M), aripiprazole once-monthly (AOM) and other oral antipsychotics (OAP) in clinical practice

**DOI:** 10.1192/j.eurpsy.2021.1438

**Published:** 2021-08-13

**Authors:** L. Gutiérrez Rojas, S. Sánchez Alonso, M. García Dorado, P. Lopez

**Affiliations:** 1 Department Of Psychiatry., University of Granada., Granada, Spain; 2 Psychiatry, Fundación Jiménez Díaz, Madrid, Spain; 3 Medical Affairs, Janssen-Cilag Spain, Madrid, Spain

**Keywords:** Relapse prevention, schizophrénia, Antipsychotics, Long-acting Antipsychotics

## Abstract

**Introduction:**

It has been shown that long-acting treatments can significantly improve adherence, control symptom, and reduce the risk of relapse compared to oral drugs. However, limited real world evidence is available as to whether there are differences among the various formulations marketed.

**Objectives:**

This study aims to assess the impact on several prognosis variables of PP1M,PP3M,AOM and OAP drugs.

**Methods:**

All adults (≥18 years) with schizophrenia who were initiated on PP1M, PP3M, AOM, or OAP treatment (chlorpromazine,levomepromazine,fluphenazine,haloperidol,ziprasidone,zuclopenthixol,olanzapine,quetiapine,asenapine,amisulpride, risperidone,aripiprazole,paliperidone) between 2017 and 2018 were identified in IQVIA’s database(1.8M of inhabitants from 4 Spanish areas). The rate of hospitalizations, emergency room visits, and treatment persistence was calculated using the Kaplan-Meier method. Survival curves were compared using the log-rank test (Sidak-adjustment),and Cox´s Hazard Ratios (HR) were used for the comparison between groups.

**Results:**

Data from 2275 patients were analyzed (PP1M= 387;PP3M=490;AOM=75;OAP=1323).The mean age of patients was 46.8(14.95) years, and 62.9% were male. The hospitalization rate at 12 months was significantly lower (p<0.01) for PP3M (8.3%) than for AOM (21.2%), PP1M (22.1%),and OAP (29.4%).When compared with PP3M use, the HRs were 2.17 for PP1M, 2.22 for AOM,and 2.90 for OAP. Emergency room visits rate at 12 months was also significantly lower (p<0. 01) for PP3M (23%) than for PP1M (36.9%), OAP (43.5%),and AOM (46.2%). Persistence rates were higher for PP3M (91%) than for any other treatment (p<0.01).

**Conclusions:**

Our results outline that patients treated with PP3M experienced fewer relapses and decompensations compared to all other treatments analyzed, which might help improve the prognosis and quality of life of patients.

**Conflict of interest:**

This study was sponsored by Janssen. M. García and P. López are employees of Janssen.

